# Patterns of cigarette and e-cigarette use among UK adolescents: a latent class analysis of the Millennium Cohort Study

**DOI:** 10.1093/eurpub/ckad124

**Published:** 2023-08-12

**Authors:** Charlotte Vrinten, Jennie C Parnham, Márta K Radó, Filippos T Filippidis, Hanna Creese, Nicholas S Hopkinson, Anthony A Laverty

**Affiliations:** Public Health Policy Evaluation Unit, School of Public Health, Imperial College London, London, UK; Public Health Policy Evaluation Unit, School of Public Health, Imperial College London, London, UK; Department of Medical Epidemiology and Biostatistics, Karolinska Institutet, Stockholm, Sweden; Public Health Policy Evaluation Unit, School of Public Health, Imperial College London, London, UK; Child Health Unit, School of Public Health, Imperial College London, London, UK; National Heart and Lung Institute, Imperial College London, Royal Brompton Hospital Campus, London, UK; Public Health Policy Evaluation Unit, School of Public Health, Imperial College London, London, UK

## Abstract

**Background:**

Patterning of cigarette and e-cigarette use among young people remains poorly characterized. We aimed to describe these patterns in the UK Millennium Cohort Study at age 14 and 17 years.

**Methods:**

Data on cigarette and e-cigarette use come from 9731 adolescents. Latent class analysis assigned participants to membership of classes of product use and multinomial logistic regression analyses assessed differences in the likelihood of belonging to classes by sociodemographic (age, gender, ethnicity, household income, maternal education and country of residence) and smoking-related social factors (caregiver tobacco use, caregiver e-cigarette use and peer smoking).

**Results:**

We identified four classes of use: 45.8% of adolescents ‘continued to abstain’ from cigarettes or e-cigarettes; 21.3% ‘experimented’ (used once or in the past but not currently) with cigarettes and/or e-cigarettes by age 17 but were not current users; 19.0% were ‘late adopters’, characterized by low levels of use at age 14 but high levels of experimentation and current use at age 17; and 13.9% were ‘early adopters’, characterized by high levels of experimentation and current use at ages 14 and 17. At age 17, 70.4% of ‘early adopters’ smoked cigarettes regularly plus an additional 27.3% experimented with cigarettes. Corresponding percentages for e-cigarettes were 37.9% and 58.9%. Tobacco and e-cigarette use by caregivers, and cigarette use by peers, were associated with being both ‘late adopters’ and ‘early adopters’.

**Conclusions:**

Approximately one in seven adolescents in the UK are ‘early adopters’ of nicotine products. This highlights the need to develop and implement effective policies to prevent nicotine use uptake.

## Introduction

The majority of people who smoke tobacco start smoking before they reach adulthood, with adolescence in particular being a time of experimentation.^1^^,^^2^ The emergence of novel nicotine and tobacco products in recent years has disrupted the tobacco market in the UK and worldwide.^3^ Previous studies in samples of UK adolescents have shown that by late adolescence about half have ever tried smoking cigarettes with ∼6% of 11–18 year olds smoking cigarettes in the last 30 days.^2^^,^^4–6^ Over 40% of 11–18 year olds have ever tried an e-cigarette, with ∼5% reporting use at least once per month.^4–8^ Recent trends show that the use of e-cigarettes in this age group is increasing, while the prevalence of cigarette use is declining.^4^^,^^6^

Risk factors for product use in adolescence appear similar for both cigarettes and e-cigarettes and include use of these products by family members or peers^2^^,^^5^^,^^8^^,^^9^ and increasing age.^5^^,^^7^^,^^8^ UK studies have found an association between lower socioeconomic status and tobacco use, with less consistent patterns for e-cigarettes.^2^^,^^4^^,^^8^ What is less well studied is the patterning of the use of these products across the population. Adolescents often experiment with different products and may use cigarettes and e-cigarettes concurrently, making this a complex picture that requires differentiated data on usage patterns with implications for both research and policy.

Latent class analysis (LCA) is a useful tool when there are complex patterns of behaviours. It is a data-driven dimension reduction technique that clusters individuals into unobserved (i.e. *latent*) classes based on their observed responses to a set of categorical variables.^10^ LCA identifies subgroups with common response patterns for further analysis and is especially helpful for combining data with many possible response combinations. LCA analyses have, for example, characterized patterns of tobacco use across different products and changes over time^11^^,^^12^ and identified disparities in the use of multiple tobacco products.^13^ Such analyses, however, have not been conducted in the UK which is an important case study with low smoking rates and regulations for e-cigarettes which are relatively permissive e-cigarette. UK authorities have encouraged switching to e-cigarette use as a means to support smoking cessation. Although in the UK e-cigarettes are subject to specific regulations regarding licensing and nicotine content as set out by the EU Tobacco Products Directive, which are not in place in other jurisdictions such as the US,^14^ enforcement of these remains incomplete. For example, there have been substantial cuts to Trading Standards officers over the last decade and illicit and non-compliant products are widely available. There is concern that this combination may be associated with greater and earlier use among UK adolescents.

The aim of this study was to describe the patterns of cigarette and e-cigarette use among UK adolescents. In particular, we (i) use latent class analysis to identify distinct classes of cigarette and e-cigarette users and examine the prevalence of these classes of use in a cohort of UK adolescents and (ii) use multinomial regression to examine the sociodemographic and smoking-related social factors associated with class membership.

## Methods

### Participants

We analysed data from the UK Millennium Cohort Study (MCS), a longitudinal cohort study of nearly 19 000 children born in the UK between September 2000 and January 2002.^15^ The study used a stratified, clustered, random sampling method with intentional oversampling in disadvantaged and ethnically diverse areas, as well as the smaller nations of the UK.^15^ All families with children registered for universal child benefits and turning 9 months old during the survey period in sample wards were invited to participate. The response rate among eligible families was 72.0%, with a survey response of 76.3% of eligible families at wave 6 (age 14) and 74.6% at wave 7 (17 years).^15^^,^^16^ All waves of the MCS received Research Ethics approvals and all respondents provided informed consent to participate.^17^

Data for the MCS are collected from both cohort members and their caregivers. We used data collected from children and the main caregiver at wave six (collected in 2015 when children were ∼14 years old) and wave seven (collected in 2018 when children were ∼17 years old). We refer to these here as baseline (age 14) and follow-up (age 17).

### Measures

#### Cigarette use

Cigarette use at age 14 and age 17 was assessed with a single question asking cohort members ‘which one of the following statements best describes you?’ with response options (1) ‘I have never smoked cigarettes’, (2) ‘I have only ever tried smoking cigarettes once’, (3) ‘I used to smoke sometimes but I never smoke a cigarette now’, (4) ‘I sometimes smoke cigarettes now but I don’t smoke as many as one a week’, (5) ‘I usually smoke between one and six cigarettes a week’ and (6) ‘I smoke more than six cigarettes a week’. We collapsed these into three categories of ‘Never’ (1) ‘Experimented but not regular use’ (2–3) and ‘Current smoking’ (4–6).

#### E-cigarette use

E-cigarette use at age 14 was assessed by asking cohort members which of four statements best described them: (1) ‘I’ve never used or tried electronic cigarettes (e-cigarettes)’, (2) ‘I have used e-cigarettes but don’t at all now’, (3) ‘I now smoke e-cigarettes occasionally but not every day’, and (4) ‘I smoke e-cigarettes every day’. E-cigarette use at age 17 was assessed by asking respondents which of six statements best described them: (1) ‘I have never tried an e-cigarette or vaping device’, (2) ‘I have only ever tried an e-cigarette or vaping device once’, (3) ‘I used to use an e-cigarette or vaping device sometimes but I never use an e-cigarette or vaping device now’, (4) ‘I sometimes use an e-cigarette or vaping device now but I don’t use an e-cigarette or vaping device as often as once a week’, (5) ‘I usually use an e-cigarette or vaping device between one and six times a week’, (6) ‘I usually use an e-cigarette or vaping device more than six times a week’. Similar to cigarette use, we recoded responses into three categories: ‘Never’ (response option 1 for both waves), ‘Experimented but not regular use’ (option 2 at age 14; options 2–3 at age 17), and ‘Current use’ (options 3–4 at age 14; options 4–6 at age 17).

#### Sociodemographic factors

We included a range of sociodemographic factors: age at baseline, gender, ethnicity, household income, maternal education and UK country of residence. We collapsed ages 14 and 15 years due to small numbers of 15-year-olds. Ethnicity was recorded in six groups and recoded for analysis into ‘White’ vs. ‘Black and Minority Ethnic (BAME)’ due to low numbers in some of the ethnic minority groups. Household income at baseline was based on the Organisation for Economic Co-operation and Development (OECD) equivalized income and was categorized into quintiles.^18^ Highest maternal educational qualification at birth was classified into four categories: ‘degree level or above’ (higher education), ‘A-levels or diplomas’ (secondary or further education exams generally taken at age 18), ‘GCSE or O-levels’ (secondary education exams generally taken at age 16), and ‘None or other qualifications’.

#### Smoking-related social factors

We also used data on exposure to smoking and e-cigarettes in the social environment. We used main caregiver current tobacco use at baseline assessed with the question ‘Do you use tobacco products such as cigarettes, cigars, a pipe or chewing tobacco at all these days?’, categorized as ‘yes’, ‘no’ and ‘no answer’. Main caregiver current e-cigarette use was assessed in the questionnaire completed by the caregiver at follow-up by asking the question: ‘How often do you currently use an e-cigarette or vaping device?’ The response options ‘daily’, ‘less than daily, but at least once a week’, ‘less than weekly, but at least once a month’ and ‘less than monthly’ were recoded for analyses as ‘Yes’, while the response ‘not at all’ was recoded as ‘No’. Peer smoking was assessed at baseline by asking cohort members: ‘How many of your friends smoke cigarettes? Do not include e-cigarettes’ with the response option ‘none of them’ recoded for analysis as ‘No’, while the responses ‘some of them’, ‘most of them’ and ‘all of them’ were recoded for analysis as ‘Yes’. For these variables, missing, ‘don’t know’, and ‘I do not wish to answer’ responses were coded as a separate category.

### Analyses

We included all cohort members who had participated in both waves six and seven of data collection, excluding those with missing all data on product use. We first provided unweighted descriptive statistics in absolute numbers and weighted percentages. We then fit a series of latent class models to the cigarette and e-cigarette use variables using the *gsem* command in Stata. This estimated missing data on tobacco and e-cigarette use with maximum likelihood estimation. As our interest was in patterns of cigarette and e-cigarette use in adolescence (rather than transitions over time), we analysed baseline and follow-up together rather than changes between these. We determined the optimal number of latent classes to describe the patterns of cigarette and e-cigarette use in this cohort of adolescents based on goodness-of-fit according to the log-likelihood, Akaike’s Information Criterion (AIC) and Bayesian Information Criterion (BIC). We ran 10 iterations of the model with the optimal number of classes to ensure convergence at a global optimum. We used survey weights from age 14, provided by the survey team to adjust for non-response bias, sampling and attrition.^15^^,^^16^^,^^18^ We then calculated the predicted posterior class probability and used this to assign each cohort member to one class. We conducted unadjusted and adjusted multinomial logistic regression analyses to determine associations between sociodemographic and smoking-related social factors and class membership. Multinomial regression is an extension of binary logistic regression analysis and is suitable for categorical dependent variables with more than two levels.^19^

## Results

Of the 9848 cohort members, we excluded 117 (1.2%) with missing data on all four cigarette and e-cigarette use variables, leaving a total analytical sample of *N* = 9731. Most participants (74.9%) were 14/15 years old at baseline, with an equal gender distribution (49.5% male) (table 1). Tobacco use at baseline was reported by 18.7% of main caregivers and 6.1% used e-cigarettes at follow-up. Tobacco smoking among peers at follow-up was reported by 29.6% of participants, with a further 10.1% not answering this question.

**Table 1:** Sample characteristics, including cigarette and e-cigarette use (unweighted, *N* = 9731)

	*N* (%)
Age at baseline (years)	
13	2446 (25.1)
14/15	7285 (74.9)
Gender	
Male	4814 (49.5)
Female	4917 (50.5)
Ethnicity	
White	7711 (79.2)
BAME	2020 (20.8)
Household income quintile	
Q1 (lowest)	1546 (15.9)
Q2	1532 (15.7)
Q3	1962 (20.2)
Q4	2312 (23.8)
Q5 (highest)	2379 (24.5)
Highest maternal education	
Degree or above	1978 (20.3)
A-levels or diplomas	1871 (19.2)
GCSE or O-levels	3855 (39.6)
None or other qualifications	1657 (17.0)
Missing	370 (3.8)
Country	
England	6502 (66.8)
Wales	1299 (13.4)
Scotland	1021 (10.5)
Northern Ireland	909 (9.3)
Caregiver tobacco use at baseline	
No	7835 (80.5)
Yes	1815 (18.7)
Missing	81 (0.8)
Caregiver e-cigarette use at follow-up	
No	6617 (68.0)
Yes	598 (6.1)
Missing	2516 (25.9)
Peer cigarette smoking at baseline	
No	5873 (60.3)
Yes	2877 (29.6)
Missing	981 (10.1)
Adolescent cigarette use at baseline	
Never	8214 (84.4)
Experimented but not regular use	962 (9.9)
Current use	320 (3.3)
Missing	235 (2.4)
Adolescent e-cigarette use at baseline	
Never	8135 (83.8)
Experimented but not regular use	1100 (11.3)
Current use	252 (2.6)
Missing	226 (2.3)
Adolescent cigarette use at follow-up	
Never	5183 (53.3)
Experimented but not regular use	2181 (22.4)
Current use	1812 (18.6)
Missing	555 (5.7)
Adolescent e-cigarette use at follow-up	
Never	4608 (47.4)
Experimented but not regular use	3518 (36.2)
Current use	1049 (10.8)
Missing	556 (5.7)

BAME, Black and Minority Ethnic.

### Cigarette and e-cigarette use at age 14 and age 17

At age 14, 9.9% of participants had tried cigarettes but were not actively smoking, while 3.3% reported current cigarette use (table 1). These numbers rose to 22.4% having tried cigarettes at age 17 with 18.6% reporting current use. At age 14, 11.3% reported having tried e-cigarettes but not using them now, while 2.6% reported current use of e-cigarettes. These numbers rose to 36.2% for trying e-cigarettes and 10.8% for current use of e-cigarettes at age 17.

### Latent class analyses

The fit statistics indicated that the model with four classes had the best relative fit (Supplementary table S1). We present the four classes found in table 2. Class 1 (‘continued abstainers’, 45.8% of the sample), consisted of respondents with a low likelihood of cigarette and e-cigarette use at baseline as well as follow-up. Class 2 (‘experimenters’, 21.3% of the sample) is characterized by a low likelihood of reporting current cigarette or e-cigarette use at baseline, but a high likelihood of reporting having tried them, combined with low current use at follow-up. Class 3 (‘late adopters’, 19.0% of the sample) is characterized by a low likelihood of having tried or using cigarettes or e-cigarettes at baseline but a relatively high likelihood of having tried or using them at follow-up. Finally, class 4 (‘early adopters’, 13.9% of the sample) is characterized by a high likelihood of experimentation (∼50%) or current use (29% for cigarettes, 21% for e-cigarettes) at baseline and a high likelihood of current cigarette (70%) and e-cigarette use (38%) at follow-up.

**Table 2:** Prevalence and item response probabilities for the 4-class model and suggested interpretations of the classes (‘class names’; weighted; *N* = 9618)

Class	Class 1	Class 2	Class 3	Class 4
Class name	‘Continued abstainers’	‘Experimenters’	‘Late adopters’	‘Early adopters’
Prevalence (%)	45.8	21.3	19.0	13.9
95% CI^a^ (%)	25.9–67.1	5.4–56.4	12.7–27.6	10.4–18.3
Item response distributions (%)				
Cigarette use at baseline^b^				
Never	98.7	87.6	94.7	22.6
Experimented but not regular use	1.3	12.2	5.3	48.3
Current use	<0.1	0.2	<0.1	29.1
E-cigarette use at baseline				
Never	99.9	81.1	91.9	25.9
Experimented but not regular use	<0.1	18.5	8.1	53.4
Current use	<0.1	0.4	<0.1	20.7
Cigarette use at follow-up^b^				
Never	93.2	38.8	12.6	2.3
Experimented but not regular use	5.7	58.0	26.0	27.3
Current use	1.1	3.2	61.4	70.4
E-cigarette use at follow-up				
Never	88.8	24.1	10.0	3.2
Experimented but not regular use	11.2	75.7	54.5	58.9
Current use	<0.1	0.2	35.5	37.9

a 95% confidence interval.

b Baseline, approximately at age 14 years; follow-up, approximately at age 17 years.

Current cigarette and e-cigarette use was virtually absent among the ‘continued abstainers and experimenters’ at baseline (<1%), although the likelihood of reporting e-cigarette use was slightly higher at follow-up (1–3%) for ‘continued abstainers’. The ‘experimenters’ group was more likely than the ‘continued abstainers’ to try cigarettes or e-cigarettes, especially at follow-up (58.0% and 75.5%, respectively). Both the ‘late adopters’ and ‘early adopters’ groups had a high likelihood of reporting cigarette and e-cigarette use at follow-up (61.4% reported cigarette use and 35.5% e-cigarette use for ‘late adopters’, and 70.4% and 37.9% for ‘early adopters’, respectively). The ‘early adopters’ were more likely to use cigarettes and e-cigarettes at baseline (29.1% and 20.7%, respectively) than ‘late adopters’ (both <0.1%).

### Sociodemographic and smoking-related social environment variables associated with class membership

Adolescents who were older at baseline (14/15 years vs. 13 years) were more likely to be ‘early adopters’ [odds ratio (OR) = 1.43, 95% confidence interval (CI) 1.23–1.67] (table 3). Girls were less likely to be ‘late adopters’ than boys (OR = 0.88, 95% CI 0.78–0.99), although there were no other differences by gender. Those whose mothers had GCSE or O-level qualifications were more likely to be ‘experimenters’ than those whose mothers had a degree or higher qualification, while those with mothers with no qualifications were more likely to be ‘early adopters’ (OR = 1.73, 95%CI 1.40–2.13). Adolescents from Scotland were more likely to be ‘late adopters’ than adolescents from England, while those from Northern Ireland were more likely to be ‘experimenters or late adopters’.

**Table 3:** Unadjusted multinomial regression analysis of associations between sociodemographic and smoking-related social factors, and four patterns of cigarette and e-cigarettes use (*N* = 9731)

	Class 1	Class 2 vs. Class 1		Class 3 vs. Class 1		Class 4 vs. Class 1	
	‘Continued abstainers’	‘Experimenters’ vs. ‘Continued abstainers’		‘Late adopters’ vs. ‘Continued abstainers’		‘Early adopters’ vs. ‘Continued abstainers’	
	*N* (%)	*N* (%)	OR (95% CI)	*N* (%)	OR (95% CI)	*N* (%)	OR (95% CI)
Age at baseline							
13	1504 (26.5)	391 (25.0)	Ref	311 (24.3)	Ref	240 (20.1)	Ref
14/15	4181 (73.5)	1176 (75.0)	1.08 (0.95; 1.23)	971 (75.7)	1.12 (0.98; 1.29)	957 (79.9)	1.43 (1.23; 1.67)
Gender							
Male	2793 (49.1)	753 (48.1)	Ref	669 (52.2)	Ref	599 (50.0)	Ref
Female	2892 (50.9)	814 (51.9)	1.04 (0.93; 1.17)	613 (47.8)	0.88 (0.78; 0.99)	598 (50.0)	0.96 (0.85; 1.09)
Ethnicity							
White	4248 (74.7)	1393 (83.2)	Ref	1128 (88.0)	Ref	1032 (86.2)	Ref
BAME	1437 (25.3)	264 (16.8)	0.60 (0.52; 0.69)	154 (12.0)	0.40 (0.34; 0.48)	165 (13.8)	0.47 (0.40; 0.56)
Household income							
Q1 (lowest)	910 (16.0)	230 (14.7)	Ref	175 (13.7)	Ref	231 (19.3)	Ref
Q2	816 (14.4)	279 (17.8)	1.35 (1.11; 1.65)	198 (15.4)	1.26 (1.01; 1.58)	239 (20.0)	1.15 (0.94; 1.41)
Q3	1106 (19.4)	324 (20.7)	1.16 (0.96; 1.40)	274 (21.4)	1.29 (1.05; 1.59)	258 (21.6)	0.92 (0.75; 1.12)
Q4	1367 (24.1)	389 (24.8)	1.13 (0.94; 1.35)	299 (23.3)	1.14 (0.93; 1.40)	257 (21.5)	0.74 (0.61; 0.90)
Q5 (highest)	1486 (26.1)	345 (22.0)	0.92 (0.76; 1.11)	336 (26.2)	1.18 (0.95; 1.44)	212 (17.7)	0.56 (0.46; 0.69)
Maternal education							
Degree or higher	1239 (22.7)	280 (18.5)	Ref	280 (22.6)	Ref	179 (15.7)	Ref
A-levels or diplomas	1141 (20.9)	289 (19.0)	1.12 (0.93; 1.35)	244 (19.7)	0.95 (0.78; 1.14)	197 (17.3)	1.20 (0.96; 1.49)
GCSE or O-levels	2101 (38.5)	691 (45.5)	1.46 (1.25; 1.70)	543 (43.8)	1.14 (0.97; 1.34)	520 (45.6)	1.71 (1.43; 2.06)
Other or none	981 (17.9)	258 (17.0)	1.16 (0.96; 1.41)	173 (13.9)	0.78 (0.63; 0.96)	245 (21.5)	1.73 (1.40; 2.13)
Country							
England	3861 (67.9)	1036 (66.1)	Ref	792 (61.8)	Ref	813 (67.9)	Ref
Wales	751 (13.2)	196 (12.5)	0.97 (0.82; 1.15)	183 (14.3)	1.19 (0.99; 1.42)	169 (14.1)	1.07 (0.89; 1.28)
Scotland	571 (10.1)	167 (10.7)	1.09 (0.91; 1.31)	154 (12.0)	1.31 (1.08; 1.60)	129 (10.8)	1.07 (0.87; 1.32)
Northern Ireland	502 (8.8)	168 (10.7)	1.25 (1.03; 1.50)	153 (11.9)	1.49 (1.22; 1.81)	86 (7.2)	0.81 (0.64; 1.04)
Caregiver tobacco use at baseline							
No	4855 (85.4)	1222 (78.0)	Ref	998 (77.9)	Ref	760 (63.5)	Ref
Yes	776 (13.7)	329 (21.0)	1.68 (1.46; 1.94)	281 (21.9)	1.76 (1.51; 2.05)	429 (35.8)	3.53 (3.07; 4.06)
Missing	54 (0.9)	16 (1.0)	1.18 (0.67; 2.06)	3 (0.2)	0.27 (0.08; 0.87)	8 (0.7)	0.95 (0.45; 2.00)
Caregiver e-cigarette use at follow-up							
No	4021 (70.7)	1053 (67.2)	Ref	852 (66.5)	Ref	691 (57.7)	Ref
Yes	253 (4.5)	123 (7.9)	1.86 (1.48; 2.33)	95 (7.4)	1.77 (1.38; 2.27)	127 (10.6)	2.92 (2.33; 3.67)
Missing	1411 (24.8)	391 (24.9)	1.06 (0.93; 1.21)	335 (26.1)	1.12 (0.97; 1.29)	379 (31.7)	1.56 (1.36; 1.80)
Peer tobacco smoking at baseline							
No	4176 (73.4)	774 (49.4)	Ref	710 (55.4)	Ref	213 (17.8)	Ref
Yes	920 (16.2)	612 (39.1)	3.59 (3.16; 4.08)	416 (32.5)	2.66 (2.31; 3.06)	929 (77.6)	19.80 (16.78; 23.35)
Missing	589 (10.4)	181 (11.5)	1.66 (1.38; 1.99)	156 (12.2)	1.56 (1.28; 1.89)	55 (5.6)	1.83 (1.34; 2.49)

BAME, Black and Minority Ethnic.

Markers of social exposure to smoking were all related to class membership. Main caregiver tobacco use was associated with being more likely to be in any of the user classes than the non-user class (‘experimenter’ OR = 1.68, 95% CI 1.46–1.94; ‘late adopter’ OR *=* 1.76, 95% CI 1.51–2.05; ‘early adopter’ OR = 3.53, 95% CI 3.07–4.06) than children whose main caregiver did not use tobacco. Similarly, main caregiver e-cigarette use was associated with being more likely to be in classes 2–4 (‘experimenter’ OR = 1.86, 95%CI 1.48–2.33; ‘late adopter’ OR = 1.77, 95% CI 1.38–2.27; ‘early adopter’ OR = 2.92, 95% CI 2.33–3.67) than children with main caregivers who did not use e-cigarettes. Peer tobacco smoking was also associated with being more likely to be in one of classes 2–4; most strongly for being an ‘early adopter’, e.g. OR = 19.80, 95% CI 16.78–23.35.

In adjusted models, girls were more likely to be ‘early adopters’ (Adjusted odds ratio [adjusted odds ratio (AOR)] = 1.30, 95%CI 1.00–1.70) than boys. BAME children were less likely to be ‘late adopters’ (AOR = 0.58, 95% CI 0.46–0.74), or ‘early adopters’ (AOR = 0.57, 95% CI 0.38–0.96) than White children (table 4).

**Table 4:** Adjusted multinomial regression analysis of associations between sociodemographic and smoking-related social factors, and four patterns of cigarette and e-cigarette use (*N* = 9731)

	Class 2 vs. Class 1	Class 3 vs. Class 1	Class 4 vs. Class 1
	‘Experimenters’ vs. ‘Continued abstainers’	‘Late adopters’ vs. ‘Continued abstainers’	‘Early adopters’ vs. ‘Continued abstainers’
	AOR (95% CI)	AOR (95% CI)	AOR (95% CI)
Age at baseline			
13	Ref	Ref	Ref
14/15	1.04 (0.87; 1.23)	1.01 (0.85; 1.20)	1.30 (1.00; 1.70)
Gender			
Male	Ref	Ref	Ref
Female	0.90 (0.76; 1.06)	0.96 (0.83; 1.12)	0.92 (0.73; 1.16)
Ethnicity			
White	Ref	Ref	Ref
BAME	0.91 (0.73; 1.12)	0.58 (0.46; 0.74)	0.57 (0.38; 0.86)
Household income			
Q1 (lowest)	Ref	Ref	Ref
Q2	1.04 (0.74; 1.44)	1.42 (1.03; 1.97)	1.51 (0.93; 2.44)
Q3	1.13 (0.78; 1.63)	1.08 (0.79; 1.49)	1.13 (0.69; 1.85)
Q4	0.86 (0.62; 1.20)	1.20 (0.88; 1.64)	1.12 (0.70; 1.80)
Q5 (highest)	1.00 (0.72; 1.39)	1.10 (0.80; 1.51)	1.06 (0.65; 1.72)
Maternal education			
Degree or higher	Ref	Ref	Ref
A-levels or diplomas	1.06 (0.85; 1.31)	0.94 (0.76; 1.18)	0.85 (0.61; 1.18)
GCSE or O-levels	1.02 (0.83; 1.27)	0.89 (0.72; 1.09)	1.01 (0.74; 1.38)
Other or none	0.91 (0.67; 1.23)	0.93 (0.69; 1.27)	1.48 (0.93; 2.33)
Country			
England	Ref	Ref	Ref
Wales	0.93 (0.72; 1.18)	1.30 (0.96; 1.76)	0.80 (0.58; 1.12)
Scotland	0.94 (0.75; 1.18)	1.02 (0.84; 1.24)	1.15 (0.86; 1.54)
Northern Ireland	1.24 (0.96; 1.60)	1.38 (1.12; 1.71)	1.65 (0.92; 2.99)
Caregiver tobacco use at baseline			
No	Ref	Ref	Ref
Yes	1.06 (0.82; 1.38)	1.46 (1.16; 1.84)	2.13 (1.57; 2.90)
Missing	0.93 (0.32; 2.65)	0.39 (0.13; 1.18)	0.64 (0.12; 3.54)
Caregiver e-cigarette use at follow-up			
No	Ref	Ref	Ref
Yes	1.44 (1.01; 2.07)	1.65 (1.20; 2.28)	1.76 (1.15; 2.68)
Missing	1.02 (0.79; 1.33)	1.09 (0.90; 1.33)	1.56 (1.16; 2.10)
Peer tobacco smoking at baseline			
No	Ref	Ref	Ref
Yes	2.89 (2.30; 3.63)	3.74 (3.09; 4.52)	27.49 (20.75; 36.42)
Missing	1.16 (0.91; 1.49)	1.49 (1.19; 1.86)	2.96 (1.80; 4.85)

AOR, adjusted odds ratio; 95% CI, 95% confidence interval; BAME, Black and Minority Ethnic.

Measures of social exposure to smoking were also linked to class membership in these adjusted models. Children with a main caregiver who used tobacco were more likely ‘late adopters’ (AOR = 1.46, 95%CI 1.16–1.84) or ‘early adopters’ (AOR = 2.13, 95%CI 1.57–2.90) than children with a main caregiver who does not smoke tobacco. Children with caregivers who used e-cigarettes were more likely to be in one of the classes 2–4 than be ‘continued abstainers’. For example, they were more likely to be ‘early adopters’ (AOR = 1.76, 95% CI 1.15–2.68). Children whose main caregiver did not have data on e-cigarette use were more likely to be ‘early adopters’ (AOR = 1.56, 95% CI 1.16–2.10) than those whose main caregiver reported no e-cigarette use. Reporting peer smoking was associated with being in one of classes 2–4, most strongly for being an ‘early adopter’ (AOR = 27.49, 95% CI 20.75–36.42), than those reporting that their peers do not smoke cigarettes.

## Discussion

This analysis is the first to use latent class methods to characterize both tobacco and e-cigarette use among UK children. It identifies four probabilistic classes of children: ‘continued abstainers’, who used neither product; ‘experimenters’ who report low levels of irregular use; ‘late adopters’, with low levels of product use at age 14 but more use at age 17; and ‘early adopters’ who report high levels of use at both ages 14 and 17 years. It finds that the largest group of children were ‘continued abstainers’. Approximately one in five children were in each of the ‘experimenters’ or ‘late adopters’ classes, while around one in seven were ‘early adopters’. Caregiver tobacco use, caregiver e-cigarette use and tobacco smoking among peers were all associated with being an ‘early adopter’.

Our finding that a large proportion of children used neither tobacco nor e-cigarettes between the ages of 14 and 17 is consistent with existing literature. For example, previous evidence reviews and empirical research have identified that about half of adolescents have not used e-cigarettes.^4^ Much of the existing use of latent class models is from the USA. For example, an analysis of more than 77 000 US adolescents identified three latent classes of tobacco use, with the majority (90%) belonging to the minimal/non-use class, similar to our classes of continued abstainers and experimenters combined.^13^ Analyses of city-dwelling US adolescents found that 40% were not at risk of tobacco use, 24% had ever used a tobacco product and 10% used more than one tobacco product.^20^ In our sample, we found higher levels of e-cigarette experimentation than cigarette experimentation, but low levels of current use, which fits with existing UK evidence that there are high levels of experimentation that do not translate into sustained use.^21^^,^^22^

We found that approximately one in seven children in the UK were ‘early adopters’. Among this class, 58.1% were dual users of cigarettes and e-cigarettes (defined as experimenting with and/or currently using both products) at age 14. This rose to 88.4% at age 17, with 28.2% of adolescents in this class currently using both products. This is a concern as we know that early use of tobacco is linked to greater use and health risks, although evidence on e-cigarette use at young ages is scarce.^1^^,^^23^ The majority of research in the area has focused on whether e-cigarettes act as a gateway into tobacco use, finding that this may be the case, although with a high degree of publication bias.^5^^,^^24^ This finding of a class of children using both products in a sustained way from an early age suggests that rather than a gateway effect there may be dual susceptibility in some groups of children from an early age.

We find that children exposed to tobacco smoking and nicotine use in their social environment are more likely to adopt e-cigarette and tobacco smoking themselves, in line with current evidence.^2^^,^^25^^,^^26^ In particular, the impact of tobacco smoking among peers was strongly associated with being an ‘early adopter’.^9^ These findings are a reminder of the need to act at a number of levels to tackle the harms of tobacco and the use of e-cigarettes by children. Interventions such as increasing enforcement of age of sale regulations would be useful, as well as raising legal age of sale and more innovative ideas such as the use of a ‘polluter pays’ levy to force the tobacco industry to pay for the measures needed to reduce and eliminate tobacco smoking.^27^

There are strengths and limitations to this work. We used a large sample designed to be representative of the population of the UK making our findings generalizable to the wider population. Survey weighting designed to account for differential attrition between groups. We also used a data-driven analysis method to group children into probabilistic classes, rather than relying on *a priori* categorizations. LCA delivers interpretable groupings of response patterns, but does lead to some heterogeneity of responses within classes. For example, some adolescents who do not smoke at age 14 are nevertheless classified as members of the ‘early adopters’ class based on their responses to the other variables. In addition, because of the probabilistic nature of class assignment, there can be some uncertainty around the class assignment, which can manifest itself as wider confidence intervals for class prevalence, such as for the ‘experimenters’ class. Nevertheless, the average posterior class probabilities, a measure of classification uncertainty, met the conventionally used minimum cut-off value of 0.70 to indicate well separated classes and adequate class assignment accuracy for all classes.^10^ These findings should be interpreted that we are presenting the most likely class membership for individuals. We combined the LCA with multinomial regression to provide insight on who is more likely to be in each of these classes. This approach allowed the assessment of associations between socio-demographics and product use among the children themselves as well as in their social environment. Nonetheless, there were limitations, many in relation to the use of secondary data which had already been collected. The questions used to assess e-cigarette use changed slightly between baseline and follow-up, which may have affected responses. Our measures of exposure to smoking in the social environment were only available at one time point each: main caregiver and peer cigarette use were only available at baseline, while main caregiver e-cigarette use was only available at follow-up. In particular, no data were available on peer e-cigarette use which is an avenue for future research. There was considerable non-response from main caregivers, especially at follow-up, meaning that we may have underestimated the proportion of product use among them.^28–30^ We used two different measures of socio-economic status (household income and maternal educational qualifications) and it is possible that other measures or conceptualizations of this construct would produce different findings.^31^ For product use, we categorized responses as never; experimentation; and current and were unable to use a more granular assessment of frequency. This work covers data from 2014 until 2018—the market for both tobacco and e-cigarettes has changed over time.^6^^,^^32^ Further research could conduct such analyses on more up to date data, as well as assessing other possible influences including perceptions of harm and marketing.^33–35^

## Conclusion

This analysis finds that although large numbers of UK children used neither tobacco nor e-cigarettes, approximately one in seven are ‘early adopters’, characterized by high levels of use at both ages 14 and 17 years. This is more likely among those with tobacco and e-cigarette use by caregivers, and particularly those reporting tobacco smoking among peers.

## Supplementary Material

ckad124_Supplementary_Data

## Data Availability

The data used for this study are available from the UK Data Service: https://ukdataservice.ac.uk. University College London, UCL Institute of Education, Centre for Longitudinal Studies. (2023). Millennium Cohort Study. [data series]. 15th Release. UK Data Service. SN: 2000031, http://doi.org/10.5255/UKDA-Series-2000031.
