# Supplementary material for: Patterns of cigarette and e-cigarette use among UK adolescents: a latent class analysis of the Millennium Cohort Study
Source: Eur J Public Health. 2023 Aug 12;33(5):857–63. doi: 10.1093/eurpub/ckad124 (PMC10567249; doi:10.1093/eurpub/ckad124)
Supplement: ckad124_Supplementary_Data [file ckad124_supplementary_data.pdf]

Appendix. Patterns of cigarette and e-cigarette use among UK adolescents: a latent class analysis of the Millennium Cohort Study

Charlotte Vrinten, Jennie Parnham, Marta Rado, Filipos Filippidis, Hanna Creese, Nicholas Hopkinson, Anthony Laverty

Supplementary table 1: Latent class analysis model fit statistics with 1 to 5 classes based on cohort member's use of cigarettes and e-cigarettes at age 14 and at age 17 (N=12,153).

No. of classes	Log likelihood	df	AIC	BIC
1	-30484.48	8	60984.96	61044.2
2	-26800.14	17	53634.28	53760.17
3	-26289.72	26	52631.43	52823.97
4	-26142.95	35	52355.90	52615.08
5[$]	-26245.06	35	52560.13	52819.32

[$]Non-converging model; maximum iterations reached (n=300).
